# Quantifying the risk of local Zika virus transmission in the contiguous US during the 2015–2016 ZIKV epidemic

**DOI:** 10.1186/s12916-018-1185-5

**Published:** 2018-10-18

**Authors:** Kaiyuan Sun, Qian Zhang, Ana Pastore-Piontti, Matteo Chinazzi, Dina Mistry, Natalie E Dean, Diana Patricia Rojas, Stefano Merler, Piero Poletti, Luca Rossi, M Elizabeth Halloran, Ira M Longini, Alessandro Vespignani

**Affiliations:** 1 Laboratory for the Modeling of Biological and Socio-technical Systems, Northeastern University, Boston, 02115 USA; 2 Department of Biostatistics, College of Public Health and Health Professions, University of Florida, Gainesville, 32611 USA; 3 Bruno Kessler Foundation, 38123 Trento, Italy; 4 Institute for Scientific Interchange Foundation, 10126 Turin, Italy; 5 Vaccine and Infectious Disease Division, Fred Hutchinson Cancer Research Center, Seattle, 98109 USA; 6 Department of Biostatistics, University of Washington, Seattle, 98195 USA

**Keywords:** Zika virus, Risk assessment, Computational modeling

## Abstract

**Background:**

Local mosquito-borne Zika virus (ZIKV) transmission has been reported in two counties in the contiguous United States (US), prompting the issuance of travel, prevention, and testing guidance across the contiguous US. Large uncertainty, however, surrounds the quantification of the actual risk of ZIKV introduction and autochthonous transmission across different areas of the US.

**Methods:**

We present a framework for the projection of ZIKV autochthonous transmission in the contiguous US during the 2015–2016 epidemic using a data-driven stochastic and spatial epidemic model accounting for seasonal, environmental, and detailed population data. The model generates an ensemble of travel-related case counts and simulates their potential to have triggered local transmission at the individual level in the 2015–2016 ZIKV epidemic.

**Results:**

We estimate the risk of ZIKV introduction and local transmission at the county level and at the 0*.*025° × 0*.*025° cell level across the contiguous US. We provide a risk measure based on the probability of observing local transmission in a specific location during a ZIKV epidemic modeled after the epidemic observed during the years 2015–2016. The high spatial and temporal resolution of the model allows us to generate statistical estimates of the number of ZIKV introductions leading to local transmission in each location. We find that the risk was spatially heterogeneously distributed and concentrated in a few specific areas that account for less than 1% of the contiguous US population. Locations in Texas and Florida that have actually experienced local ZIKV transmission were among the places at highest risk according to our results. We also provide an analysis of the key determinants for local transmission and identify the key introduction routes and their contributions to ZIKV transmission in the contiguous US.

**Conclusions:**

This framework provides quantitative risk estimates, fully captures the stochasticity of ZIKV introduction events, and is not biased by the under-ascertainment of cases due to asymptomatic cases. It provides general information on key risk determinants and data with potential uses in defining public health recommendations and guidance about ZIKV risk in the US.

**Electronic supplementary material:**

The online version of this article (10.1186/s12916-018-1185-5) contains supplementary material, which is available to authorized users.

## Background

From 2015 to 2016, the Zika virus (ZIKV) epidemic spread across most countries in the Americas, including the United States (US) [1–3]. As of July 3, 2018, three US territories, including Puerto Rico, have reported 37,255 ZIKV cases mostly due to widespread local transmission [3, 4]. Laboratory evidence of possible ZIKV infections has been found in 4900 pregnant women from US territories, 167 of whom have had pregnancy outcomes with ZIKV-related birth defects [3, 5, 6]. The US states and District of Columbia have reported 5710 travel-associated ZIKV cases, including 2474 pregnant women with evidence of ZIKV infection and 116 ZIKV-related birth defects [3]. Two geographical locations have experienced local transmission of ZIKV in the contiguous US: Miami-Dade County, in Florida, and Cameron County, in Texas [7, 8]. While the outbreaks in Florida and Texas were limited, the indirect impact on the local economy has been remarkable [9].

Concerns have been raised that several other locations in the contiguous US were at risk of ZIKV transmission, thus triggering a number of studies aimed at identifying populations at highest risk of local transmission [10–20]. In particular, detailed studies based on environmental suitability, epidemiological factors, and travel-related case importations have been used to estimate the risk for specific counties in the US [21, 22]. In this study, we quantify the risk of local ZIKV transmission by using a data-driven stochastic and spatial epidemic model accounting for seasonal, environmental, and detailed population data. The model also accounts for the association between socioeconomic status and the risk of exposure to mosquitoes, and it has been previously used to estimate the introduction of Zika in the Americas and the spatial and temporal dynamics of the epidemic [23]. By using an extensive likelihood analysis with data from places with a reliable epidemiological surveillance system, the model generates a stochastic ensemble of simulations estimating the place and time of introduction of ZIKV in Brazil and the unfolding of the epidemic in the Americas. For each simulation, the individual-level scale of the model allows for the construction of daily travel-related case counts (TCCs) tracking the number of infected individuals in the contiguous US at the county level and at the finer spatial resolution of 0*.*025° × 0*.*025° corresponding approximately to 2*.*5 km × 2*.*5 km cells, comparable in size to the ZIKV active transmission areas identified in Florida by the Centers for Disease Control and Prevention (CDC) [24]. Using the time series of county-specific TCCs and the mechanistic transmission model, it is possible to estimate the probability that a specific location would experience local ZIKV transmission during the 2015–2016 time window. The methodology proposed here provides a statistical estimate of ZIKV transmission risk that is not biased by the under-ascertainment of infections and the single historical occurrence of the case importation timeline that fail to account for the full stochasticity of transmission. The TCC database also allows us to identify key sources and routes of ZIKV introductions. Results from our study can provide guidance to public health agencies in their efforts to identify populations and seasons at high risk of ZIKV transmission, so that resources towards outbreak prevention and response can be allocated more efficiently.

## Methods

**Fig. 1:**
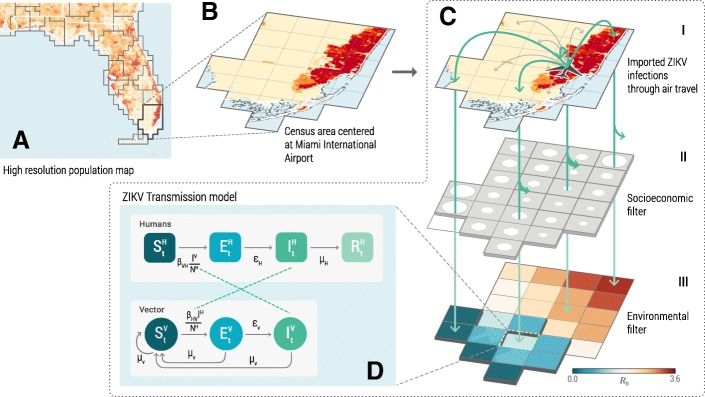
A schematic illustration of the computational framework to assess the risk of ZIKV introductions into the contiguous US. **a** High-resolution (0*.*025° × 0*.*025°∼2*.*5 km × 2*.*5 km) population density map [59] and Voronoi tessellation of the contiguous US into census areas with a major airport transportation hub at each of their centers [60]. **b** An example of the census area centered at Miami International Airport. **c** I: Travel-associated ZIKV infections entering the Miami International Airport. Location of residence of each ZIKV infection is randomly assigned with likelihood proportional to the population density within each census area. II: The probabilistic filter of the risk of exposure to mosquitoes due to socioeconomic factors such as housing conditions, sanitation, and disease awareness. III: Spatiotemporal specific ZIKV transmission dynamics are influenced by environmental factors that are temperature sensitive, including the spatial distribution of *Aedes* mosquitoes, seasonal mosquito abundance, and ZIKV transmissibility. **d** Compartmental stochastic ZIKV transmission model used to evaluate the environmental suitability of ZIKV transmission. Humans are divided into susceptible *S*^*H*^, exposed *E*^*H*^, infectious *I*^*H*^, and recovered *R*^*H*^ compartments, and mosquitoes are divided into susceptible *S*^*V*^, exposed *E*^*V*^, and infectious *I*^*V*^ compartments

We consider three major factors associated with local ZIKV transmission in the contiguous US: the intensity of travel-related infection importations, the environmental suitability for ZIKV transmission, and the socioeconomic risk of exposure to mosquitoes. In this study, we develop a data-driven computational framework (Fig. 1) to quantitatively account for these three factors and to evaluate their impact on ZIKV transmission. Based on this framework, we assess the risk of local ZIKV transmission across the contiguous US through the full course of the 2015–2016 ZIKV epidemic.

The starting point of our methodology is the construction of a synthetic database of TCC entering the US through airport transportation hubs. The database is generated from simulations based on a large-scale spatial model simulating the 2015–2016 ZIKV epidemics, where both symptomatic and asymptomatic ZIKV infections are considered [23]. The synthetic database of TCC contains for each infected individual the time of arrival, stage of ZIKV infection, airports of origin and arrival, and location of residence in the contiguous US^1^ [25]. A schematic sample of the database is shown in Table 1.

**Table 1:** A sample of the database containing simulated travel-related ZIKV-infected individuals entering the US

Case ID	Time of arrival	Airport of arrival	Stage of infection	Airport of origin	Location of residence (latitude, longitude)
0001	2015-12-01	MIA	Exposed	BOG	(25.864, − 80.257)
0101	2016-07-15	JFK	Infectious	SJU	(40.729, − 73.991)
0212	2016-11-23	MIA	Infectious	SJU	(25.808, − 80.130)

Each infected individual’s likelihood of exposure to mosquito bites and his/her capability of triggering local ZIKV transmission is affected by the ecological presence of mosquitoes in his/her location of residence. Indeed, our model integrates mosquito abundance data (*Ae. aegypti* and *Ae. albopictus*) [26, 27] that takes into account for temperature suitability, precipitation, vegetation, and urbanization and considers seasonal variations in the mosquito density determined by daily temperature. The individual’s socioeconomic status, which is strongly associated with factors such as sanitation conditions, accessibility to air conditioning, and level of disease awareness, also affects the likelihood of exposure to mosquitoes [14, 28, 29]. Our computational framework considers a data layer based on global socioeconomic indicators [30], which is calibrated with historical mosquito-borne disease outbreaks in naive populations to provide a likelihood map of the individual’s exposure to mosquitoes [23]. This map serves as a spatial filter (Fig. 1c-II) that probabilistically selects individuals exposed to mosquito bites down to the resolution of a 0*.*25° × 0*.*25° cell containing his/her location of residence. Each of the exposed individuals can potentially trigger detectable local ZIKV transmissions (Fig. 1c-III, d), according to the stochastic mechanistic ZIKV transmission model that takes into account mosquito abundance, the current temperature in the area, and the transmission dynamics of ZIKV (see Additional file 1: Supplementary Information). We define a detectable local transmission as the generation of 20 or more autochthonous transmission infections triggered by a single ZIKV infection introduction. Smaller outbreaks would likely go unnoticed assuming a 5% to 10% detection rate of infections due to the large proportion of asymptomatic cases [31–33]. Due to fine spatial and temporal resolution, the transmission model is able to account for the significant variability in the ZIKV basic reproduction number (*R*_0_) across locations, as well as the variability within the same location at different times. These differences in *R*_0_ are driven by temperature and the mosquito abundance, among other variables. The details of the mechanistic model and the calculation of the socioeconomic risk of exposure to mosquitoes are reported in Additional file 1. More technically, we can define the following procedure:

1. We randomly sample one out of the simulated TCC from the statistical ensemble output of the ZIKV model [23].
2. For each infected individual in the TCC, we stochastically determine whether he/she is potentially exposed to mosquito bites based on the probability of exposure *p*_*e*_ at the location of residence *x*. *p*_*e*_ is calibrated based on socioeconomic indicators and *x* identifies a specific county or spatial cell. In each location *x*, these individuals could potentially trigger local transmission.
3. Based on the individual’s stage of infection (exposed or infectious), time of introduction, and location of residence (at 0*.*025° × 0*.*025° resolution), we simulate local ZIKV transmission with the same stochastic transmission model used in the global model (described in Additional file 1: Supplementary Information) with the specific parameters calibrated to each 0*.*25° × 0*.*25° cell in the US.
4. For each simulated TCC, the above procedure identifies all the infections triggering detectable local transmission. For every time interval ∆*t* and geographical area *x* of interest, we can associate variable *n*(*x*, ∆*t*) = 1 if there is at least one imported infection from the TCC that triggers detectable local transmission, and *n*(*x*, ∆*t*) = 0 otherwise.

In order to provide a probabilistic risk measurement, we execute *N* = 10^6^ resamplings from the ensemble of simulated TCC generated by the model and repeat the above procedure. The resampling procedure accounts for the many possible TCCs compatible with the observed ZIKV epidemic and stochastic effects in the local transmission. This is because not all case importations will result in local outbreaks, even in areas where transmission is favored. The risk of local ZIKV transmission for area *x* during time window ∆*t* can be thus defined as

1 $$ {r}_{tr}\left(x,\Delta  t\right)=\frac{1}{N}\sum \limits_{i=1}^N{n}_i\left(x,\Delta  t\right) $$

where *i* indexes the 10^6^ outcomes from the resampled TCCs. This definition of the risk can be aggregated at various spatial (0.025° × 0.025°) and temporal resolutions (≥ 1 day), and it can be used to generate risk maps of ZIKV introduction across the contiguous US. Unless otherwise specified, we consider in this study the local transmission risk *r*_*tr*_(*x*) that is defined on the ∆*t* referring to the time window spanning from January 1, 2015 to December 31, 2016. This definition of risk can be interpreted as the probability of observing a detectable local transmission in a specific area per ZIKV epidemic.

## Results

**Fig. 2:**
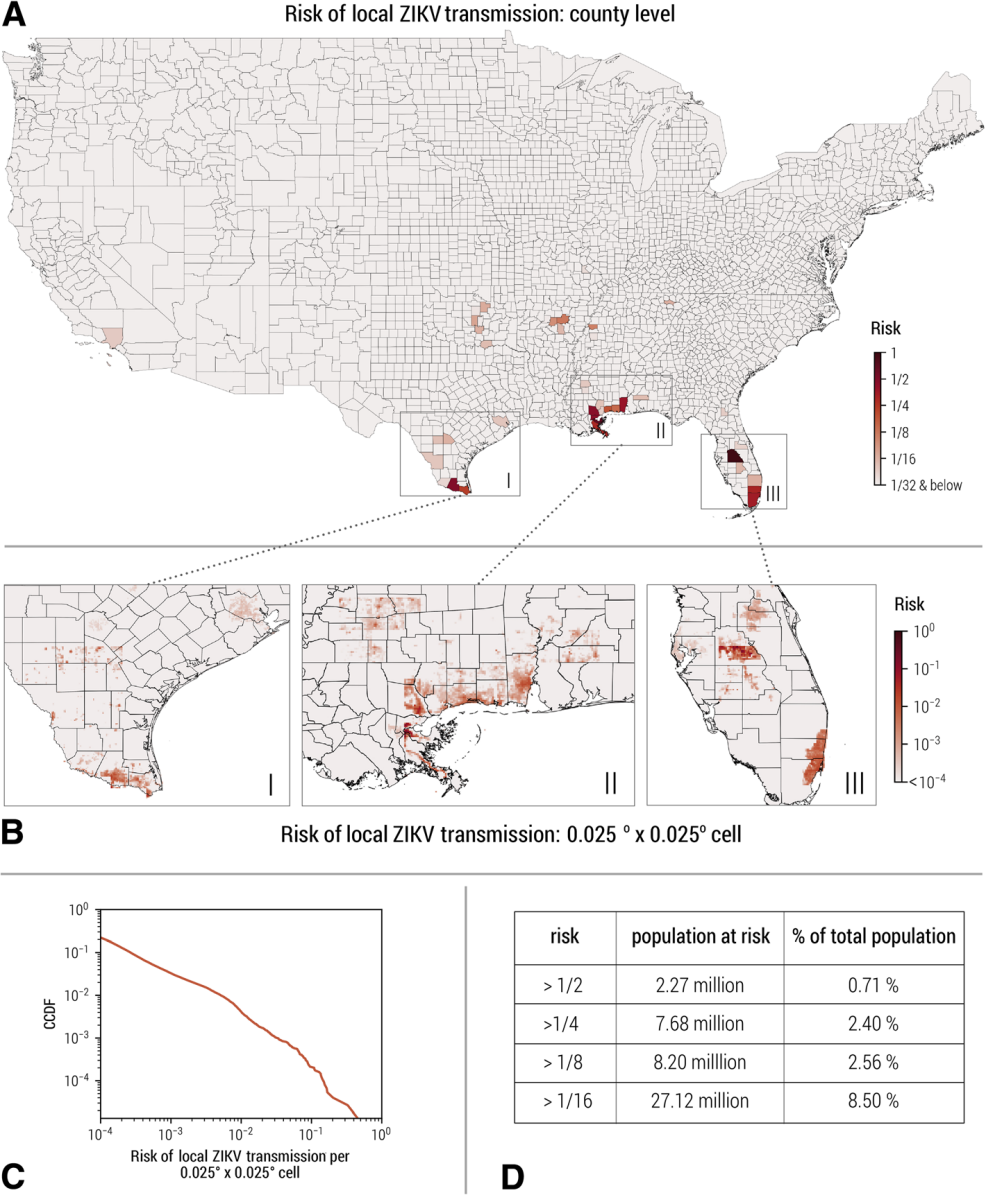
The cumulative risk of local ZIKV transmission in the contiguous US. The cumulative risk of local ZIKV transmission at different spatial resolutions is evaluated through the full course of the simulated 2015–2016 ZIKV epidemic. **a** The cumulative risk map of local ZIKV transmission for each county in the contiguous US. The color scale indicates for any given county the probability of experiencing at least one ZIKV outbreak with more than 20 infections (details in Additional file 1). **b** High spatial resolution estimates (0*.*025° × 0*.*025°) of the cumulative risk of local ZIKV transmission through the full course of the simulated 2015–2016 ZIKV epidemic. **c** The complementary cumulative distribution function of the local ZIKV transmission risk for all 0*.*025° × 0*.*025°cells (on a log-log scale). The heavy tail feature of the distribution reflects strong spatial heterogeneity in terms of local ZIKV transmission risk. **d** The total population in the counties of the US with different risk levels of local ZIKV transmission and their percentage with respect to the total population in the contiguous US

By using the methodology outlined in the previous section, we provide quantitative estimates of *r*_*tr*_(*x*) both at the county level and at 0*.*025° × 0*.*025° cell resolution. Figure 2a shows the risk of ZIKV introduction at county level in the contiguous US through the full course of the simulated 2015–2016 ZIKV epidemics. We consider four main brackets for the risk and the associated population sizes. At the county level, the highest risk bracket *r*_*tr*_(*x*) *>* 0*.*5 includes only 0.71% of the total population in the contiguous US. In these areas, one would expect to observe detectable local transmission events with a probability above 50% during the simulated 2015–2016 ZIKV epidemic. Even when we extend the high-risk bracket to include counties with *r*_*tr*_(*x*) *>* 1/8, this includes only 2.56% of the total population in the contiguous US. Thus, the risk of local transmission is extremely concentrated to specific geographical locations. Figure 2d shows the population living in counties with different risk brackets of ZIKV introduction and their percentage with respect to the total population in the contiguous US.

The counties of Miami-Dade, Florida, and Cameron, Texas, where local transmission was observed in the year 2016, were both estimated to be high-risk locations (risk bracket, greater than 1*/*4). Densely populated areas along the Gulf Coast also show up as high-risk locations, in agreement with estimates from other models [12]. The risk of ZIKV introduction and local transmission *r*_*tr*_(*x*) is highly spatially heterogeneous (Fig. 2a, b). This heterogeneity persists even within the state of Florida, where most areas are estimated to be environmentally suitable for ZIKV transmission all year long [12, 34]. This is mostly because of socioeconomic and local climate heterogeneities. At a spatial granularity of 0*.*025° × 0*.*025°, it is possible to perform a statistical analysis of the risk distribution. In Fig. 2c, we report the distribution of cell-specific risks *r*_*tr*_(*x*). The distribution has a very right-skewed heavy tail extending over more than four orders of magnitude, a clear signature of the large heterogeneity of the risk in the contiguous US.

**Fig. 3:**
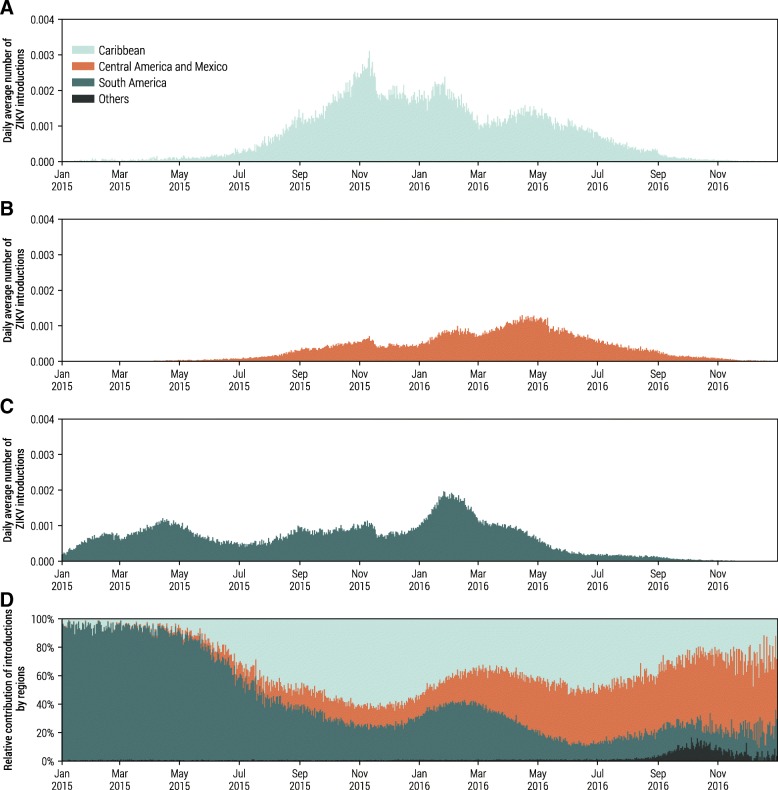
A breakdown of local ZIKV transmission events by the geographical origins of travel-associated ZIKV infections in Miami-Dade, Florida. **a**–**c** The daily average number of ZIKV imported infections per day that trigger outbreaks with more than 20 infections, originating from the Caribbean, Central America and Mexico, and South America. **d** The relative contributions to the expected number of local ZIKV transmission events by different geographical regions

It is worth stressing that the source of ZIKV introductions in each location is time-dependent, since the TCC is determined by both the magnitude of the epidemic in the regions of the Americas affected by ZIKV and travel patterns from these areas. Our model explicitly simulates individual ZIKV-infected travelers, with detailed information about the traveler’s origin and destination at the daily scale. This allows us to decompose the relative contribution of potential ZIKV introductions from different epidemic regions and to identify routes of high risk with high spatiotemporal resolution. In Table 2, we report the likelihood of local ZIKV transmission in Miami-Dade, Florida, for the year 2015 and 2016 triggered by infection importations from the Caribbean, Central America and Mexico, and South America. The likelihood accounts for intensity of ZIKV transmission in epidemic regions, the travel volume between the source regions and Miami-Dade, and the time-dependent environmental suitability of local transmission in Miami-Dade. In Fig. 3, we report the daily risk of ZIKV infections in Miami-Dade from different geographical regions as well as the time-dependent relative contributions of different regions to the risk throughout the years 2015 and 2016.

**Table 2:** The likelihood of a given local ZIKV transmission event in Miami-Dade, Florida, from different geographical regions (Caribbean, South America, Central America and Mexico) for the years 2015 and 2016

Region	Year 2015		Year 2016	
	Likelihood (%)	95% CI (%)	Likelihood (%)	95% CI (%)
Caribbean	43.81	(10.47–61.98)	40.15	(14.09–59.79)
South America	27.67	(27.87–78.42)	27.67	(16.10–47.31)
Central America and Mexico	10.50	(3.61–20.39)	30.02	(17.54–48.52)

As shown in both Table 2 and Fig. 3, in 2015, countries in the Caribbean and South America were major contributors to ZIKV introduction risk in Miami-Dade. On the other hand, countries in Central America and Mexico became major contributors in 2016. This reflects the fact that the ZIKV epidemic started earlier in South American countries, including Brazil and Colombia, and later on spread to countries in Central America and Mexico. Caribbean countries, however, remained a major source of infection importation in both 2015 and 2016. This is possibly due to the high travel volumes between Florida and the Caribbean, as well as high incidence rate and weak seasonality of ZIKV transmission in that region. This is in line with epidemiological data from Florida’s Department of Health, as well as phylogenetic analysis based on sequenced ZIKV genomes from both infected humans and mosquitoes in Florida [35].

**Fig. 4:**
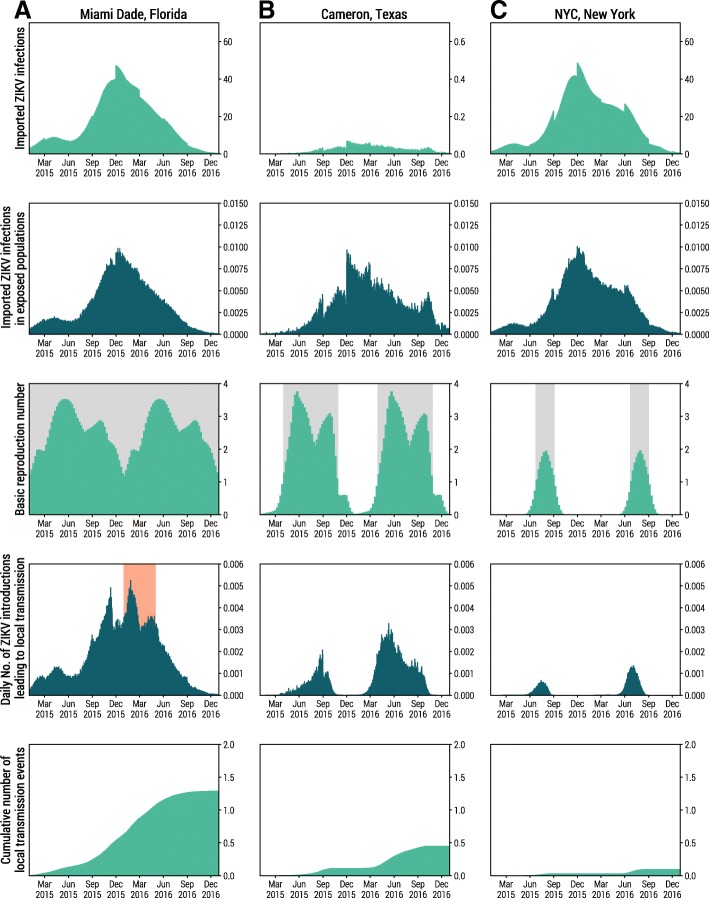
Factors which co-shape the spatiotemporal risk of local ZIKV transmission in three different regions in the contiguous US. Columns from left to right represent **a** Miami-Dade, Florida; **b** Cameron, Texas; and **c** New York City, New York. Row 1 shows the average daily number of imported ZIKV infections. Note that for Cameron, Texas, the scale on the *y*-axis is different than that of Miami-Dade, Florida, and NYC, New York. Row 2 shows the average number of imported ZIKV infections that pass through the socioeconomic filter *p*_*e*_ and reside in areas potentially exposed to mosquitoes. Row 3 shows the basic reproduction number (weekly average) calculated based on the ZIKV transmission model. Gray-shaded time windows indicate when the basic reproduction number *R*_0_ *>* 1 and sustainable ZIKV transmission is possible. Row 4 shows the expected daily number of ZIKV introductions with the red-shaded time window indicating the estimated time of local ZIKV transmission based on phylogenetic analysis [35]. Row 5 shows the average cumulative number of local ZIKV transmission events since January 1, 2015

In Fig. 4, we zoom in on three representative areas to disentangle the key determinants shaping the spatiotemporal risk of local ZIKV transmission. Panels a, b, and c in Fig. 4 represent geographical areas covering Miami-Dade, Florida; Cameron, Texas; and New York City, New York. Both Miami-Dade and New York City experienced a high volume of ZIKV infection importations due to high population density and close proximity to major international transportation hubs. Cameron, Texas, on the other hand, had far fewer ZIKV infection importations. However, due to socioeconomic factor (among other factors), the population in Cameron, Texas, is more likely to be exposed to mosquitoes than the populations of Miami-Dade and New York City. Consequently, the volume of Cameron’s imported infections that were exposed to mosquito bites is comparable to those of Miami-Dade and New York City.

The environmental suitability of ZIKV transmission in the three areas is remarkably different. The basic reproduction number *R*_0_ is above the epidemic threshold (*R*_0_ *>* 1) in Miami-Dade throughout the year, indicating ZIKV transmission is environmentally suitable all year long. Cameron, Texas, has moderate environmental suitability, where *R*_0_ drops below the threshold in winter seasons. New York City is far less environmentally suitable for ZIKV transmission, with a narrow time window of approximately 2 months during summer when *R*_0_ is larger than 1.

Given the individual-level resolution of the model, we can focus on the daily average number of travel-associated ZIKV infections leading to local transmission. This is a different indicator than risk. The latter is defined as the probability of observing at least one event of detectable local transmission in the area, thus overlooking the number of different introduction events that trigger local transmission. The profile of daily ZIKV introductions that would lead to local transmission (Fig. 4, row 4) is jointly shaped by ZIKV infection importations, socioeconomic risk of exposure to mosquitoes, and the environmental suitability of ZIKV transmission. The cumulative number of ZIKV introductions leading to local transmission was high in both Miami-Dade, Florida, and Cameron, Texas, where local transmission occurred in the year 2016. The time of ZIKV introduction in Miami-Dade, Florida, is estimated to have occurred between January and May 2016 based on phylogenetic analysis of sequenced ZIKV genomes from infected patients and *Ae. aegypti* mosquitoes [35]. Our model suggests (Fig. 4, row 4) high risk of ZIKV introduction during the same time window, despite relatively low environmental suitability. The high risk of introduction in Miami-Dade between January and May 2016 was mainly driven by a high influx of imported ZIKV infections. Based on our simulations, Miami-Dade county has on average 1.29 cumulative introductions leading to local transmission events (95%CI (0–9)) throughout 2015 and 2016 (Fig. 4, row 5, insert). However, the distribution of the number of introductions is positively skewed (skewness *γ*_1_ = 4*.*40), with a maximum of 55 introductions. This indicates the possibility of multiple introductions during the ZIKV outbreak in Miami-Dade, Florida, in line with estimates from phylogenetic analysis [35].

To investigate to what extent the spatial variation of local ZIKV transmission is driven by key socioeconomic and environmental determinants, we first consider a regression model exploring the relation between the average number of local ZIKV transmissions (log(*n*_*tr*_) is the dependent variable) and three key determinants: the number of ZIKV importations, average temperature, and the GDP per capita. Specifically, the explanatory variables include:

- log(*N*_*im*_), the logarithm of the cumulative average number of TCC for each 0*.*25° × 0*.*25° cell from January 1, 2015, to December 31, 2016.
- log(*f*_20_°), the logarithm of the fraction of days over the year with an average temperature larger than 20 °C for each 0*.*25° × 0*.*25° cell.
- log(*GDP*), the gross domestic product per capita in terms of purchasing power parity for each 0*.*25° × 0*.*25° cell.

In Table 3, we show that if all three explanatory variables are included in the regression (model 1), the model can explain 73*.*9% of the variance in the number of average introductions leading to local transmission in each cell *x*. While only considering log(*N*_*im*_) and log(*f*_20_° ) (model 2), we can explain 56*.*2% of the variance, and using log(*N*_*im*_) (model 3) alone can explain 47*.*5% of the variance. It is worth remarking that such a simple statistical analysis cannot fully explain the variance of log(*n*_*tr*_) due to the nonlinear dependency between ZIKV transmission, vector population dynamics, and temperature. It is also due to the highly nonlinear nature of the disease transmission dynamics captured by the epidemic threshold (where the basic reproduction number (*R*_0_) needs to be larger than one to be able to spread in a population). In addition, more than 90% of the geographical areas in the contiguous US are not included in the regression because the simulations project no local transmission events in those areas. However, 77% (in terms of areas) of these “risk-free” areas are not environmentally suitable for ZIKV transmission according to our model.

**Table 3:** Regression analysis between log(*n*_*tr*_) and explanatory variables including log(*N*_*im*_), log(*f*_20_° ), and log(*GDP* )

	Model 1	Model 2	Model 3
log(*N*_*im*_)	0*.*72^∗∗∗^ (0.69, 0.74)	0*.*48^∗∗∗^ (0.45, 0.51)	0*.*49^∗∗∗^ (0.46, 0.52)
log(*f*_20_°)	2*.*18^∗∗∗^ (1.89, 2.48)	3*.*40^∗∗∗^ (3.01, 3.78)	
log(*GDP*)	− 13*.*29^∗∗∗^ (− 14.20, − 12.38)		
*R* squared	74.9%	57.9%	47.5%

In model 1, all three explanatory variables log(*N*_*im*_), log(*f*_20_°), and log(*GDP*) are included. Model 2 includes log(*N*_*im*_) and log(*f*_20_° ). Model 3 only includes log(*N*_*im*_). For each model, we report the regression coefficient (95% CI) for each of the explanatory variables along with *R* squared, based on *n* = 1220 cells

*n*_*tr*_ average number of local ZIKV transmissions within each 0*.*25° × 0*.*25° cell from January 1, 2015, to December 31, 2016, *N*_*im*_ number of ZIKV importations, *f*_*20*_*°* fraction of days with temperature higher than 20 °C, *GDP* gross domestic product per capita in purchasing power parity

***p < 0.001

To better illustrate the role of the three main drivers of Zika transmission, we conduct a sensitivity analysis considering three counterfactual scenarios. In each counterfactual scenario, we modify one of the three drivers across the contiguous US to uniformly mimic the conditions in Miami-Dade, Florida, while keeping the other two drivers intact. Specifically:

- In counterfactual scenario 1, the environmental suitability (the temperature and thus all temperature-modulated disease parameters) and socioeconomic risk of exposure remain the same, while for all airports in the US, the ZIKV infection importations are set to be the same as those of the airport in Miami-Dade, Florida.
- In counterfactual scenario 2, the ZIKV infection importations and the socioeconomic risk of exposure to mosquitoes remain the same. However, in this scenario, the temperature and consequently all temperature-modulated parameters of ZIKV transmission model across the contiguous US are set to be the same as those in Miami-Dade, Florida.
- In counterfactual scenario 3, the ZIKV infection importations and the environmental suitability are kept intact, while the socioeconomic risks of exposure to mosquitoes across the contiguous US are set to be the same as that in Miami-Dade, Florida.

For each of the three counterfactual scenarios, we repeat the analysis performed with the real data and generate the cumulative county-level risk map of local ZIKV transmission during the years 2015–2016 (see Additional file 1: Supplementary Information, Section 4). All three risk maps of the counterfactual scenarios are distinctly different from the risk map of Fig. 2a. Particularly, in counterfactual scenario 1, under unrealistic high intensity of ZIKV infection importations, all areas with overlapping favorable environmental and socioeconomic determinants are at high risk of local ZIKV transmission. In counterfactual scenario 2, with unrealistic favorable environmental suitabilities of ZIKV across the US, the areas at high risk are no longer restricted to the proximity of the US southern border. Many counties with low average yearly temperature and absence of *Aedes* mosquitos in the real world present significant risks of local ZIKV transmission. In counterfactual scenario 3, with high socioeconomic status equivalent to Miami-Dade, Florida, both southern Texas and populated areas along the Gulf Coast were relieved from high probability of encountering Zika, leaving southern Florida as the only focus of high risk. Thus, all three drivers are necessarily required to evaluate the risk of local ZIKV transmission in the contiguous US.

## Discussion

A prominent feature of our findings is the spatiotemporal heterogeneity of ZIKV transmission risk across the contiguous US. Spatially, our model estimates that approximately 68.9% of the people in the contiguous US live in areas that are environmentally suitable for ZIKV transmission, in line with other models’ estimates [36]. However, taking all ZIKV introduction and transmission determinants into consideration, areas with non-negligible risk (greater than 1/8) are concentrated in densely populated areas along the Gulf Coast, capturing 2.56% of the US population. From a temporal perspective, certain areas experience strong seasonality of ZIKV environmental suitability, with a narrow time window when ZIKV transmission is possible. Given limited resources, identifying seasons and regions of high risk may help guide resource allocation for high-risk population screening, intervention, and vector control. Our model is also able to identify the high-risk routes of ZIKV importations through air travel. Imported infections originating from Caribbean countries served as a major contributor to trigger local ZIKV transmission in Florida. Although it has the highest number of estimated ZIKV infections among all countries, Brazil is not a major contributor overall (5.75% of potential introductions leading to local transmission across the contiguous US). This is due to Rio de Janeiro and Sao Paulo, two of the largest transportation hubs in Brazil which make up 65% of the international travel to US from Brazil, being located in the Southern region where ZIKV transmission activity is relatively low. In addition, Rio de Janeiro and Sao Paulo have the opposite seasonality compared to the contiguous US. When it is environmentally suitable for ZIKV transmission in Rio de Janerio and Sao Paulo, it is not suitable for ZIKV transmission in most of the US. Thus, imported ZIKV infections from Brazil were less likely to fuel potential transmissions in the US.

Our model also suggests that in Miami-Dade, Florida, the overall risk of ZIKV introduction in 2015 is comparable to that in 2016, while local transmission is only observed in 2016. This could be explained by the stochasticity of transmission events. Another possibility is that because of the high asymptomatic rate of ZIKV infections, limited local transmission events occurred in 2015 without being picked up by the surveillance system. Awareness of ZIKV was low in 2015 as the World Health Organization declared ZIKV as a Public Health Emergency of International Concerns only in early 2016. Around the same time, the CDC announced a Health Alert Network advisory for Zika virus [3], marking the start of active monitoring of ZIKV activities in the US.

The proposed model has several limitations. The high volume of cruise ship stops along coastal areas of Florida to the Caribbean may elevate the risk of ZIKV transmissions beyond what is estimated in our model. Sexual transmission and transmission through other routes, not considered by our model, may facilitate the risk of local transmission even further. From January 1, 2015, to August 9, 2017, there were 49 reported ZIKV cases in the contiguous US acquired through other routes, including sexual transmission [3, 37–39]. This indicates that a larger population may be affected by ZIKV [40–42]. In addition, ZIKV RNA was detected in semen as long as 92 days after symptom onset and is able to be sexually transmitted 31–42 days after symptom onset [43]. ZIKV’s ability to persist in infected males and the potential to infect through sexual transmission long after symptom onset are troublesome. However, the specific risk through sexual transmission or other transmission routes are not well understood, and the overall impact of ZIKV infections acquired through other routes remains unclear. As such, we do not include them in our study [44]. Risk of exposure to mosquitoes associated with socioeconomic factors is widely recognized but poorly quantified. In our model, we utilize seroprevalence studies from nine chikungunya outbreaks on confined, naive populations to estimate this association, in line with other approaches used to estimate the ZIKV attack rate [14]. Further studies however are needed to advance our understanding of the association between risk of exposure to mosquitoes and socioeconomic status.

Our model assumes the mosquito abundance is explicitly modulated by temperature, since many studies suggest that temperature is the main driver of the seasonal variation of mosquito abundance [45–48]. The effect of rainfall as an environmental driver is indirectly included into our model through incorporating the mosquito presence data created by Kraemer et al. [27]. The study suggests that for both *Ae. aegypti* and *Ae. albopictus*, maximum and minimum precipitation make significant contributions to explain the spatial distribution of *Aedes* mosquitoes, consequently affecting the environmental suitability of local ZIKV transmission. However, a full mechanistic modeling of the influence of rainfall (i.e., daily timescale) on the mosquito lifecycle, while interesting, is still out of reach on a global scale. Along with rainfall, human water supplies may also affect the availability of stagnant water, especially in urban settings [45, 49]. Without controlling for the effect of human water supplies, the effect of precipitation could be positive [50–52], negative [53], or no effect at all [54, 55]. In Additional file 1: Supplementary Information, we provide a figure illustrating the seasonal abundance provided by our model.

In our model, we consider both *Ae. aegpyti* and *Ae. albopictus* as competent vectors to transmit the ZIKV. However, the competence of *Ae. albopictus* to transmit ZIKV is debated, and the notable differences in the spatial distributions of *Ae. aegpyti* and *Ae. albopictus* make it crucial for evaluating the global risk of ZIKV [27, 56]. However, these differences are less relevant when limiting the risk assessment within the spatial range of the contiguous US. This is because the geographical distribution of the environmental suitability of *Ae. aegpyti* and *Ae. albopictus* is largely overlapping within the contiguous US, based on the studies by Johnson et al. [57]. The environmental suitability distribution of *Ae. albopictus* extends a bit further north when compared to that of *Ae. aegpyti*. In the areas where only *Ae. albopictus* are present, the overall environmental suitability of ZIKV transmission is very low due to the presence of strong seasonality, and our model estimates that those areas would have minimal risk of experiencing local ZIKV transmission in the years 2015–2016 (Fig. 2).

In 2017–2018, ZIKV transmission activities in most countries throughout the Americas has plummeted [2], in agreement with model estimates [23, 58]. The risk of ZIKV introduction in the contiguous US would be expected to be negligible as imported infections triggering the local transmission would be drastically reduced. However, one should exercise caution as vector-transmitted diseases are known to show strong spatial heterogeneity and seasonality and are affected by socioeconomic factors. The stochastic nature of ZIKV transmission could leave a considerable amount of naive populations living in regions at risk of ZIKV transmission. Furthermore, expansion of the *Aedes* mosquito distribution, human migration, and shifts in socioeconomic status could lead to more populations being at risk for local ZIKV transmission. It is more likely that ZIKV transmission activities in the future may resemble the current situation of chikungunya, where transmission activities could flare up sporadically. The possible sporadic outbreaks of ZIKV would continue to pose a risk to the contiguous US, where most of the population is naive to the virus and a large fraction live in areas environmentally suitable for ZIKV transmission.

## Conclusion

In this study, we show that the overall risk of ZIKV introduction and local transmission during the 2015–2016 outbreak is jointly determined by the intensity of ZIKV importations, environmental suitability for ZIKV transmissions, and the socioeconomic risk of exposure to mosquitoes. Our estimates suggest that the risk of ZIKV introductions has a very strong spatial and temporal heterogeneity. The areas in the contiguous US at non-negligible risk (that is, greater than 1/8) only account for 2*.*6% of the total population in the contiguous US. The model is able to identify the hotspots for ZIKV introductions, and it reveals the relative contributions of ZIKV introductions from different geographical regions over time. The results of our study have the potential to guide the development of ZIKV prevention and response strategies in the contiguous US.

## Additional file

Additional file 1: Supplementary information. (PDF 5211 kb)

## Abbreviations

CDC: Centers for Disease Control and Prevention
GDP: Gross domestic product
PPP: Purchasing power parity
TCCs: Travel-related case counts
US: United States
ZIKV: Zika virus

## Footnotes

1 Although there has been reporting in the media about the traffic to and from Latin and Caribbean countries, airline traffic in 2016 has been stable with a mere 4.4% increase.
