# Supplementary material for: Quantifying the risk of local Zika virus transmission in the contiguous US during the 2015–2016 ZIKV epidemic
Source: BMC Med. 2018 Oct 18;16:195. doi: 10.1186/s12916-018-1185-5 (PMC6194624; doi:10.1186/s12916-018-1185-5)
Supplement: Supplementary file 1 — Supplementary information. (PDF 5211 kb) [file 12916_2018_1185_MOESM1_ESM.pdf]

Quantifying the risk of local Zika virus transmission in the contiguous US during the 2015-2016 ZIKV epidemic

Supplementary Information

Kaiyuan Sun[1], Qian Zhang[1], Ana Pastore-Piontti[1], Matteo Chinazzi[1], Dina Mistry[1], Natalie E. Dean[2], Diana P. Rojas[2], Stefano Merler[3], Piero Poletti[3], Luca Rossi[4], M. Elizabeth Halloran[5,6], Ira M. Longini Jr.[2], and Alessandro Vespignani[1,4]

[1]Laboratory for the Modeling of Biological and Socio-technical Systems, Northeastern University, Boston, MA, USA
[2]Department of Biostatistics, College of Public Health and Health Professions, University of Florida, Gainesville, FL, USA
[3]Bruno Kessler Foundation, Trento, Italy
[4]Institute for Scientific Interchange Foundation, Turin, Italy
[5]Vaccine and Infectious Disease Division, Fred Hutchinson Cancer Research Center, Seattle, WA, USA
[6]Department of Biostatistics, University of Washington, Seattle, WA, USA

September 6, 2018

1 Simulating the spatiotemporal dynamics of the 2015-2016 ZIKV epidemic in the Americas

1.1 Overview

To quantify the risk of local ZIKV transmission in the contiguous US, we utilize the simulation results from the study by Zhang et. al. [28] to estimate the volume of travel-related ZIKV cases (including both symptomatic and asymptomatic infections) entering the US. In addition, to evaluate the risk of triggering local transmission in the contiguous US by travel-related cases, we adopt the ZIKV transmission model (Section 1.2, 1.3) as well as the quantification of socioeconomic risk of exposure to mosquitoes (Sections 1.5) from ref. [28]. In this section, we briefly reiterate how the global epidemic and mobility model (GLEAM) was adopted to simulate the 2015-2016 ZIKV epidemic in the Americas, while details of the model can be found in ref. [28].

1.2 ZIKV transmission dynamics

Figure S1 describes the compartmental classifications used to simulate ZIKV transmission dynamics. The human-vector chain-binomial model is based on a SEIR compartmentalization of human populations and a SEI compartmentalization of vector populations [15].

Humans can be in one of four compartments: susceptible individuals (S^H) who lack immunity against infection, exposed individuals (E^H) who have acquired infection but are not yet infectious, infected individuals (I^H) who can transmit infection (and may or may not display symptoms), and removed individuals (R^H) who are no longer infected. People in the final compartment may recover and gain immunity or die. We treat the total human population size as constant, i.e., $S^H + E^H + I^H + R^H = N^H$.

The transition of people between compartments is performed stochastically, based on various biological factors. Following ref. [12], susceptible humans transit to the exposed compartment under the force of infection (λ^H), which is proportional to the rate at which a particular human is bitten by infected mosquitoes (I^V/N^V), the parameter β that accounts for the daily mosquito biting rate and the specific transmissibility of ZIKV, and the temperature dependence [17] of the mosquito-to-human probability of transmission ($\mathcal{T}_{VH}$). The temperature dependency of $\mathcal{T}_{VH}$ is given by the following equation:

$$\mathcal{T}_{VH} = 0.001044T(T - 12.286)\sqrt{32.461 - T}. \quad (1)$$

The factor k expressing the number of mosquitoes per person, and we have $N^H = N^V/k$, yielding a force of infection $\lambda^H = k\beta\mathcal{T}_{VH}\frac{I_t^V}{N^V}$. On average, individuals stay in the exposed or infectious state for the duration of the mean intrinsic latent period $\epsilon_H^{-1} = 7$ *days* or the mean infectious period $\mu_H^{-1} = 5$ *days*, respectively.

The vector population is divided into three compartments: susceptible (S^V), exposed (E^V), and infectious (I^V). The force of infection (λ^V) governing the transition rate from susceptible to exposed individuals in the vector population is proportional to the density of infectious humans (I^H/N^H). On average, mosquitoes are in the exposed state for the duration of the mean extrinsic latent period $\epsilon_V^{-1} = 8$ *days*. The average lifetime of mosquitoes μ_V^{-1} is temperature dependent and varies across spatial locations and time of the year [11]. The explicit temperature dependency of μ_V^{-1} is given by the following equation:

$$\mu_V(T) = 0.3967 - 0.03912T + 2.442 \times 10^{-3}T^2 - 7.479 \times 10^{-5}T^3 + 9.298 \times 10^{-7}T^4. \quad (2)$$

The overall mosquito population is rescaled every day based on the local temperature. The explicit temperature dependency [28] is given by the equation:

$$k \propto \exp\{-(\tilde{T} - 25)^2/50\} \quad (3)$$

where $\tilde{T} = \sum_{i=0}^{78} T(t-i)/79$ is the average temperature of the previous 79 days. Similar to the force of infection from vector to human, the force of infection from human to the vector, λ^V, is a function of β, the temperature dependence of human-to-mosquito transmission ($\mathcal{T}_{HV}$), and the density of infectious humans ($\frac{I_t^H}{N^H}$). We thus have $\lambda^V = \beta\mathcal{T}_{HV}\frac{I_t^H}{N^H}$.

The coupled population equations describing the epidemic time evolution read as:

$$
\begin{aligned}
S^H_{t+1} &= S^H_t - \Delta_{S^H \to E^H} && (4)\\
E^H_{t+1} &= E^H_t + \Delta_{S^H \to E^H} - \Delta_{E^H \to I^H} && (5)\\
I^H_{t+1} &= I^H_t + \Delta_{E^H \to I^H} - \Delta_{I^H \to R^H} && (6)\\
R^H_{t+1} &= R^H_t + \Delta_{I^H \to R^H}, && (7)
\end{aligned}
$$

and

$$
\begin{aligned}
S^V_{t+1} &= S^V_t - \Delta_{S^V \to E^V} + \Delta_{I^V \to S^V} + \Delta_{E^V \to S^V} && (8)\\
E^V_{t+1} &= E^V_t - \Delta_{E^V \to S^V} - \Delta_{E^V \to I^V} + \Delta_{S^V \to E^V} && (9)\\
I^V_{t+1} &= I^V_t + \Delta_{E^V \to I^V} - \Delta_{I^V \to S^V}. && (10)
\end{aligned}
$$

In the above expressions each term $\Delta_{X \to Y}$ represents the number of human or vector individuals transitioning from state X to state Y. Transitions are calculated according to chain binomial processes $\Delta_{X \to Y} = Binomial(X, p_{X \to Y})$, and $p_{X \to Y}$ are transition probabilities determined by the force of infection and the average lifetime of individuals in each compartment. We assume memoryless discrete stochastic transition processes. It is worth stressing that the terms $\Delta_{I^V \to S^V}$, $\Delta_{E^V \to S^V}$ are introduced to model the replenishment of mosquitoes after death.

By using the standard approach of Ref. [12], the basic reproduction number can be expressed as:

$$
R_0 = \frac{\epsilon_V}{(\epsilon_V + \mu_V)(\mu_V \mu_H)} k \beta^2 \mathcal{T}_{VH} \mathcal{T}_{HV}. \quad (11)
$$

It is worth remarking that the basic reproduction number varies spatiotemporally according to the temperature and mosquito abundance.

Figure S1: Compartmental classification for ZIKV infection. Humans can occupy one of the four top compartments: susceptible, which can acquire the infection through contacts (bites) with infectious mosquitoes; exposed, where individuals are infected but are not yet able to transmit the virus; infectious, where individuals are infected and can transmit the virus to susceptible mosquitoes; and recovered or removed, where individuals are no longer infectious. The compartmental model for the mosquito vector is shown below with similar construction to that for the human.

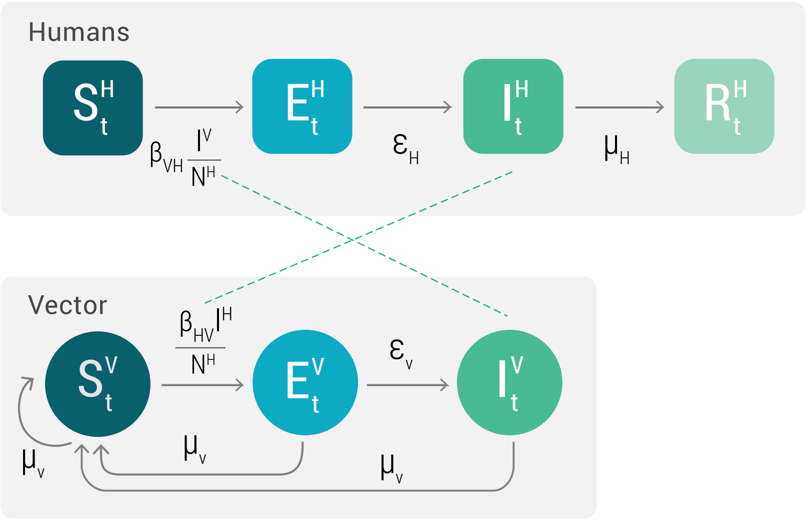

Table S1: Parameters used in the model. $T_{dep.}$ denotes parameters that are temperature dependent. $T, G_{dep.}$ denotes parameters that are temperature and geolocation dependent.

Parameter	Definition	Value	Reference
$1/\epsilon_V$	extrinsic latent period	8 days	[10, 28]
$1/\epsilon_H$	intrinsic latent period	7 days	[10, 28]
$1/\mu_H$	human infectious time	5 days	[10, 28]
ρ_a	proportion of asymptomatic cases	0.8	[7]
$1/\mu_V$	mosquito lifespan	$T_{dep.}$	[11, 28]
k	mosquitoes per person	$T, G_{dep.}$	[28]
T_{VH}	mosquito-to-human prob. of transmission	$T_{dep.}$	[17]
T_{HV}	human-to-mosquito prob. of transmission	$T_{dep.}$	[17]

1.3 The calibration of the ZIKV transmission model

The calibration of the disease dynamic model is performed by a Markov chain Monte Carlo (MCMC) analysis of data reported from the 2013 ZIKV epidemic in French Polynesia [16]. Setting the extrinsic and intrinsic latent periods and the human infectious period to reference values and using average daily temperatures of French Polynesia, we estimate a basic reproduction number at the temperature $T = 25$ C° from French Polynesia as $R_0^{FP} = 2.75$ (95% CI [2.53-2.98]), which is consistent with other ZIKV outbreak analyses [16, 5]. It is worth recalling that the reproduction number depends on the location and time of the year through seasonal temperature changes. The calibration in French Polynesia provides the basic transmissibility of ZIKV.

1.4 The projected seasonal abundance of the mosquitoes in the contiguous United States

To model the seasonal variation of the mosquito abundance across the contiguous United States, we consider a mechanistic model of mosquito population dynamics driven by local temperature [28]. The model is informed by the mosquito presence data created by Kraemer et. al [13] and the global air temperature data [27] and projects the daily abundance of *Aedes* mosquitoes at the spatial resolution of $0.25° \times 0.25°$ across the contiguous US. Figure S2 provide the projected monthly average *Ae. aegypti* abundance of 50 major cities in the contiguous US comparable to the projection by Monaghan et al. [18]. Here we consider the maximum monthly abundance in Miami as a reference point, we define abundance category "None to low" as less than 10% of the reference point, category "Low to moderate" as $10\% - 50\%$ of the reference point, "Moderate to high" as $50\% - 90\%$ of the reference point, and "High" as larger than 90% of the reference point.

1.5 The socioeconomic risk of exposure to mosquitoes

Studies suggest that even if the environmental conditions are suitable for arbovirus transmission, population's risk of exposure to mosquitoes may still vary due to socioeconomic disparities [26, 23, 24]. Following ref. [28], we estimate the socioeconomic risk, p_e throughout the contiguous US. The premise of the method is to identify the fraction of the population exposed to mosquito biting (i.e., p_e) given the presence of mosquitoes due to socioeconomic disparities. To accomplish this, we consider 9 previous outbreaks of chikungunya that occurred in confined populations (either island populations or isolated villages). We estimate the socioeconomic risk of exposure p_e for each of the 9 outbreaks considered utilizing seroprevalence surveys and environmental settings related to chikungunya transmission. We consider the logarithm of Gross Domestic Product per capita in Purchasing Power Parity (GDP per capita, PPP) as the socioeconomic indicator and model the relationship between p_e and $log(GDP\ per\ capita, PPP)$ by considering a linear regression model:

$$\hat{p}_e = \hat{\alpha} + \hat{\beta} log(GDP\ per\ capita, PPP) \quad (12)$$

Figure S2: Projected monthly abundance of *Ae. aegypti* in 50 major cities of the contiguous United States

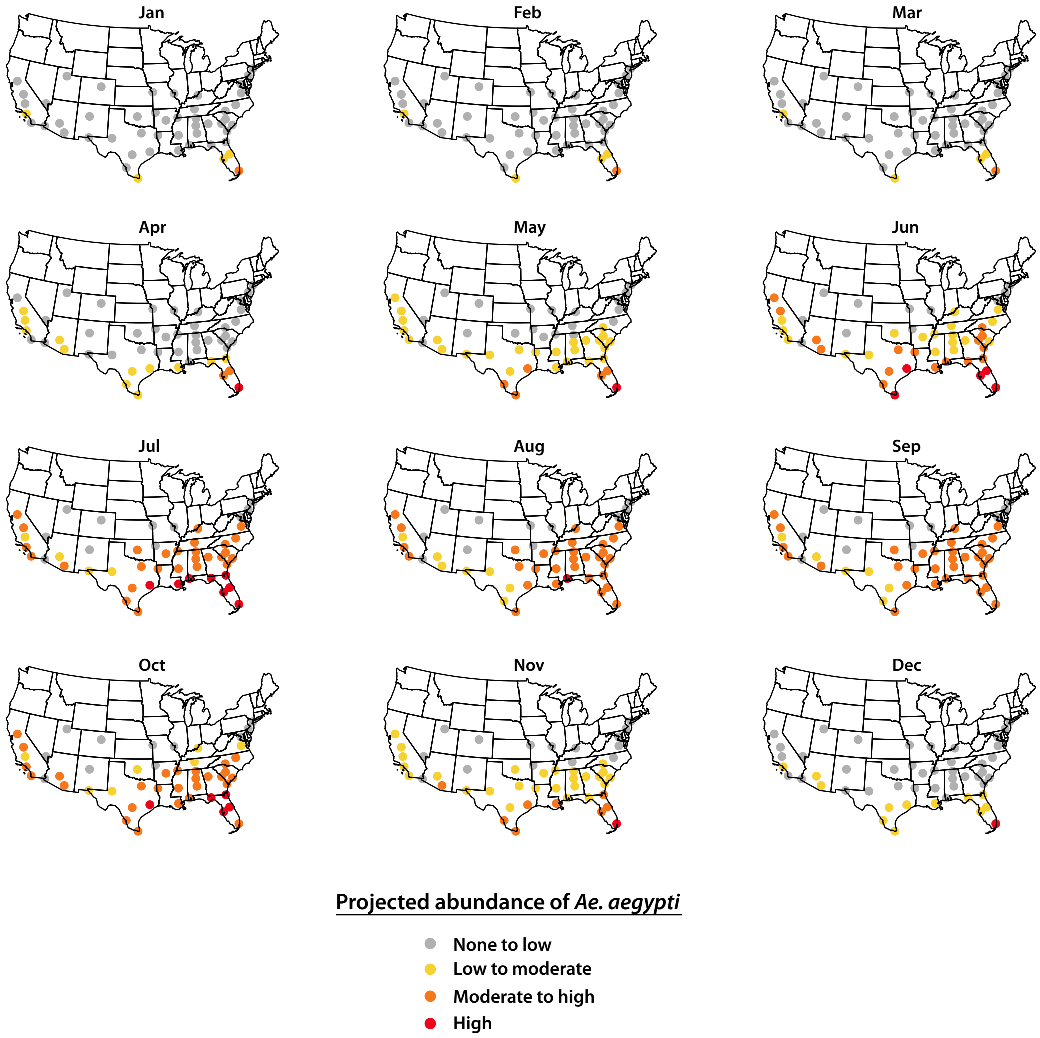

where $\hat{\alpha}$ and $\hat{\beta}$ were estimated through least squares regression. To project the socioeconomic risk of exposure $\hat{p}_e$ across the contiguous US, we consider the geophysically scaled economic dataset (G-Econ), developed by Nordhaus et al.[21, 20]. This data maps the GDP per capita in PPP at $1° \times 1°$ resolution. We spatially interpolate the data into a resolution of $0.25° \times 0.25°$, matching the spatial resolution of ZIKV transmission model (Section 3). Additionally, the data is rescaled to reflect the 2015 GDP per capita, PPP estimate.

1.6 Global model for the spread of vector-borne diseases

The GLEAM model is a fully stochastic epidemic modeling platform that utilizes real world data in order to perform *in silico* simulations of the spatial spread of infectious diseases at the global level. GLEAM uses population information obtained from the high-resolution population database of the *Gridded Population of the World* project from the Socioeconomic Data and Application Center at Columbia University (SEDAC) [6]. The model considers geographical cells of $0.25° \times 0.25°$, corresponding to an approximately $25km \times 25km$ square for cells along Earth's equator. GLEAM

groups cells into subpopulations defined by a Voronoi-like tessellation of the Earth's surface centered around major transportation hubs in different urban areas. The model includes over 3,200 subpopulations in roughly 230 different countries.

Within each subpopulation, a compartmental model is used to simulate the disease of interest. The model uses an individual dynamic where discrete, stochastic transitions are mathematically defined by chain binomial and multinomial processes. Subpopulations interact through the mechanistically simulated mobility and commuting patterns of disease carriers. Mobility includes global air travel [22], and GLEAM simulates the number of passengers traveling daily worldwide using available data on the origin-destination flows among indexed subpopulations.

The transmissibility of vector-borne diseases is associated with strong spatial heterogeneity, driven by variability and seasonality in vector abundance, the temperature dependence modulating the vector competence, and the characteristics of the exposed populations. Many locations, such as those at high elevation, are not at risk of autochthonous ZIKV transmission simply because the vector is absent. In other locations, the vector may be present but sustained transmission is not possible because of environmental factors that affect the vector's population dynamics, such as temperature or precipitation. Housing conditions, availability of air-conditioning, and socioeconomic factors also contribute significantly to determining the fraction of the population likely exposed to the vector. To extend the GLEAM model to simulate vector-borne diseases, a number of new datasets with high spatial resolution are integrated, including the following:

- *Global terrestrial air temperature data:* The global air temperature dataset [27] containing monthly mean temperatures at a spatial resolution of $0.5° \times 0.5°$. To match the spatial resolution of GLEAM's gridded population density map, the temperature for each population cell is extracted from the nearest available point in the temperature dataset. Daily average temperatures are linearly interpolated from each population's monthly averages.
- *Global Ae. aegypti and Ae. albopictus distribution:* The Global *Ae. aegypti* and *Ae. albopictus* distribution database provides uncertainty estimates for the vector's distribution at a spatial resolution of $5km \times 5km$ [13].
- *Geolocalized economic data:* The geophysically scaled economic dataset (G-Econ), developed by Nordhaus et.al. [21, 20], maps the per capita Gross Domestic Product (computed at Purchasing Power Parity exchange rates) at a $1° \times 1°$ resolution. To estimate the per capita Gross Cell Product (GCP) at Purchasing Power Parity (PPP) rates, the amount is distributed across GLEAM cells proportionally to each cell's population size. The data have also been rescaled to reflect 2015 GDP per capita (PPP) estimates.

These databases are combined to model the key drivers of ZIKV transmission. Temperature impacts many important disease parameters, including the spatiotemporally specific values of R_0, whose variations induce seasonality and spatial heterogeneity in the model. Temperature data is also used together with the mosquito presence distribution data to define the daily mosquito abundance (number of mosquitoes per human) in each cell. Data on mosquito abundance and temperature are used to identify cells where ZIKV outbreaks are not possible because of environmental factors. The human populations in these cells are thus considered unexposed to ZIKV. Finally, we use historical data and G-Econ to provide a socioeconomic rescaling factor, reflecting how exposure to the vector is impacted by socioeconomic variables such as the availability of air-conditioning.

1.7 The calibration of the global model

Once the data layers and parameters have been defined, the model runs using discrete time steps of one full day to simulate the transmission dynamic model, incorporating human mobility between subpopulations. The model is fully stochastic and from any nominally identical initialization (initial conditions and disease model), generates an ensemble of possible epidemics, as described by newly generated infections, time of arrival of the infection in each subpopulation, and the number of traveling carriers. The Latin square sampling of the initial introduction of ZIKV in the Americas, and the ensuing statistical analysis is performed on 150,000 stochastic epidemic realizations. From those realizations we find the probability $p(x)$ and $p(x|\theta)$, defined as the probability of the evidence (the epidemic peak in Colombia, as from surveillance data) and the likelihood of the evidence given the parameters θ specifying the date and location of

introduction of ZIKV in Brazil. From those distributions we can calculate the posterior probabilities of interest. In total, 1736 out of the 150,000 simulations match the epidemic peak in Colombia and the posterior estimate on the time of introduction into Brazil matches phylogenetic evidence [8, 9]. We consider the ensemble of the 1736 simulations as the output of the global model to characterize the spatiotemporal dynamics of ZIKV transmission in the Americas and the global dissemination of the travel related ZIKV infections through human mobility.

2 Challenges on using surveillance data to directly estimate the travel-associated ZIKV infections entering the contiguous US and the creation of the TCC database

Evaluating the risk of ZIKV introduction in the contiguous US requires a quantitative estimate of the intensity of travel-associated ZIKV infections entering the contiguous US. In the case of Zika, direct estimates from the surveillance data prove challenging due to the following reasons. First, the US CDC issued a health advisory for ZIKV on January 15th, 2016, before which travel associated ZIKV infections were not actively monitored in the US. However, in 2015, several countries including Brazil were likely experiencing large scale ZIKV outbreaks [28] and exporting ZIKV infected-individuals to the contiguous US which were missing from the surveillance systems. Second, a high asymptomatic rate of ZIKV infection along with mild or inapparent dengue-like disease symptoms result in low detection rate of ZIKV through surveillance system. Analysis suggests that even in the United States, the detection rate was likely lower than 5% [25], indicating that the surveillance system only revealed the tip of the iceberg of the total travel-associated ZIKV infections entering the contiguous US. Third, the publicly available data on travel-associated ZIKV infections in the contiguous US are spatially aggregated at state level and are not temporally resolved. However, since ZIKV transmission, like other vector transmitted diseases, is strongly modulated by seasonality [19], both the ZIKV infected individual's time of arrival and location of residence are important to assess his/her risk of triggering local ZIKV transmissions.

To overcome the challenge mentioned above and unveil the full scope of the ZIKV infection importations through the surveillance system, we consider the output of the computational model developed by Zhang et al. [28], and briefly reiterated in Section 1. Specifically, for each of the 1736 simulated ZIKV epidemics, we extract all the travel-associated ZIKV infections entering the US whose time and airport of entry, stage of infection (exposed or infectious), and country of origin are known. We further divide the contiguous US into census areas (referred to as airport basins hereafter) centered on airport transportation hubs using a Voronoi tessellation (details of the method can be found in [1]). For each travel-associated ZIKV infection entering the same airport basin in the US, we randomly assign his/her location of residence within the airport basin at a resolution of $2.5km \times 2.5km$. The likelihood of assignment is proportional to the population density [6] of the geographical location. This allows us to create the simulated travel associated case counts (TCC) for each of the 1736 simulations.

3 The environmental suitability of ZIKV transmission in the United States

The risk of a travel-associated ZIKV infection to trigger local transmission also varies with time and location of entry into the contiguous US. A major driver of this spatiotemporal modulation is the environmental suitability of ZIKV transmission. Previous studies [19] suggest that only limited areas surrounding the gulf coast of the contiguous US are suitable for ZIKV transmission all year long. In contrast, a large fraction of the contiguous US is not suitable for ZIKV transmission due to the limited range of the Aedes mosquitoes' spatial distribution [4, 14]. Transmission suitability in the rest of the contiguous US varies with seasonality, resulting in a transmission-allowed time window that in general decreases in length with the yearly average temperature [19, 2, 3]. When a travel-associated ZIKV infection enters the contiguous US, he/she has the potential to trigger ZIKV local transmission only during the "transmission-allowed" time window of his/her location of residence.

Figure S3: The maps ($0.25° \times 0.25°$ resolution) of basic reproduction numbers R_0s across the contiguous US on (A) March 1st, (B) June 1st, (C) September 1st, and (D) December 1st.

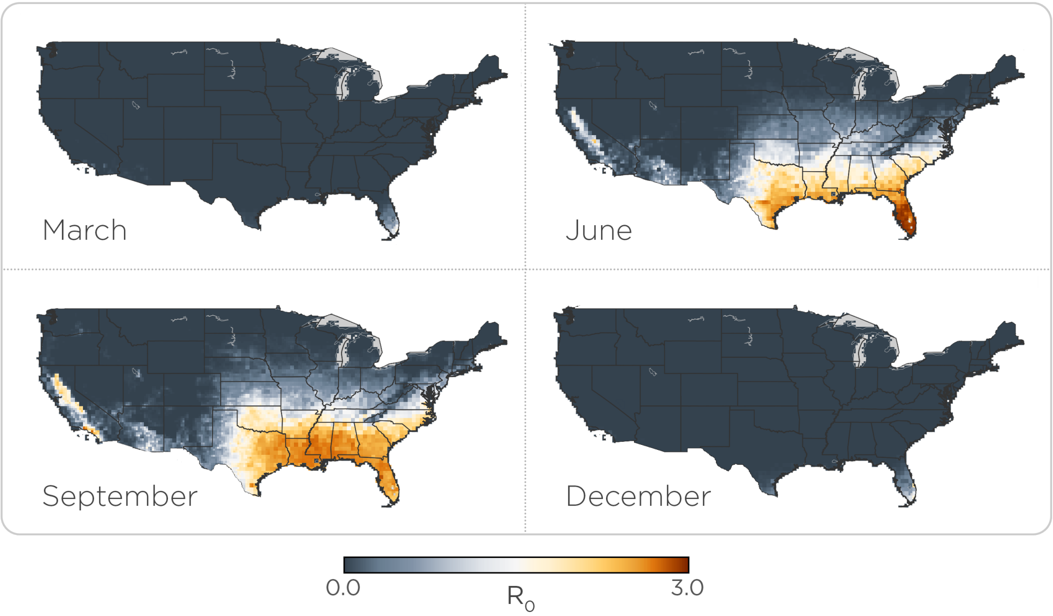

The ZIKV transmission model described in Sections 1.2 and 1.3 explicitly considers the environmental determinants of ZIKV transmission including temperature and vector distribution. Here, we inform this model with high definition data on temperature [27] and Aedes mosquito distribution [13] in the contiguous US to obtain the spatiotemporally resolved environmental suitability of ZIKV transmission. Figure S3 shows the basic reproduction number R_0 at four different seasons in the contiguous US. From the figure, we can clearly observe the strong spatiotemporal heterogeneity of the Zika virus environmental suitability in the contiguous US.

4 Sensitivity analysis and counterfactual scenarios

To illustrate that the spatiotemporal variations of the three main drivers for ZIKV transmission (i.e., environmental suitability of ZIKV transmission, the socioeconomic risk of exposure to mosquitos, and the intensity of ZIKV infection importations) give rise to the spatial heterogeneity of the risk of local ZIKV transmission, we create three counterfactual scenarios. In each scenario, we modify one of the three drivers across the contiguous United States to uniformly mimic the settings in Miami-Dade, Florida while holding the two other drivers the same as in the main text analysis (referred to as the reference scenario hereafter).

Specifically, in **Counterfactual Scenario 1**, the environmental factor (the temperature and thus all temperature-modulated disease parameters) and socioeconomic risk of exposure to mosquitoes remain the same as the reference scenario in the main manuscript, while for all airports in the US, the ZIKV infection importations are set to be the same as those of the airport in Miami-Dade, Florida. By repeating the same analysis performed with the actual data for the reference scenario, the county-level risk map of local ZIKV transmission in this scenario is shown in Figure S4.

Since the airport of Miami-Dade, Florida has one of the highest volumes of ZIKV infection importation, for areas that are environmentally suitable for ZIKV transmission, and socioeconomically at high risk of exposure to mosquitoes, the cumulative risk of local ZIKV transmission appears to be much higher when compared to the reference scenario presented in the manuscript.

Figure S4: The cumulative risk map of local ZIKV transmission for each county in the contiguous US of Counterfactual Scenario 1, where the ZIKV infection importations are set to be the same as those of the airport in Miami-Dade, Florida across all airports in the contiguous US. The color scale indicates for any given county the probability of experiencing at least one ZIKV outbreak with more than 20 infections.

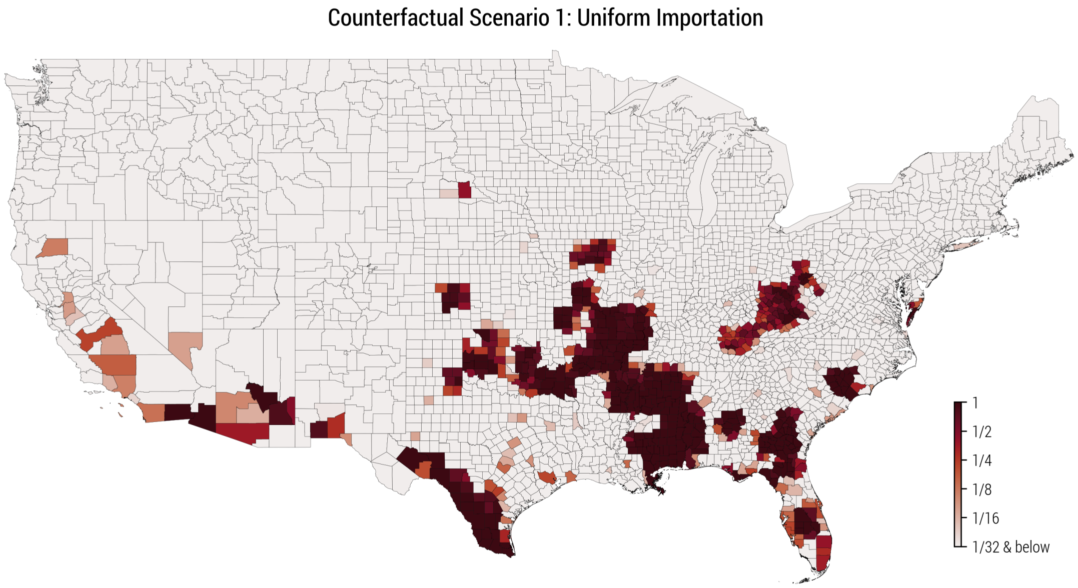

In **Counterfactual Scenario 2**, the level of ZIKV infection importation and the socioeconomic risk of exposure to mosquitoes remains the same as reference scenario. However, the temperature and consequently all temperature-modulated parameters of ZIKV transmission model across the contiguous US are set to be the same as that in Miami-Dade, Florida. The county risk map of local ZIKV transmission during the year 2015-2016 is shown in Figure S5.

In Miami-Dade, Florida, the environmental conditions favor ZIKV transmission, with the basic reproduction number R_0 greater than 1 all year long. Therefore, in this counterfactual scenario we observe that the regions at risk of local ZIKV transmission extend to multiple geographical regions previously not environmentally suitable for local ZIKV transmission ($R_0 < 1$). For instance we observe substantial risk of ZIKV local transmission in states such as Maine, New York, Illinois, Oregon, and Idaho.

In **Counterfactual Scenario 3**, the level of ZIKV infection importations and the environmental suitability (temperature and consequently temperature-modulated disease parameters) remain the same in the reference scenario. However, in this scenario, the socioeconomic risks of exposure to mosquitoes across the contiguous US are set to be the same as that in Miami-Dade, Florida. In this case the county risk map of local ZIKV transmission during the year 2015-2016 is shown in Figure S6.

Due to the low socioeconomic risk of exposure to mosquitoes in Miami-Dade, Florida in the reference scenario, in the Counterfactual Scenario 3 the only geographical location that shows substantial risk is southern Florida. In contrast, in the reference scenario areas in southern Florida, southern Texas and densely populated regions along the Gulf Coast are all areas at risk.

In summary, the simulations of all three counterfactual scenarios generate unrealistic risk maps of ZIKV local transmission across the contiguous United States. This sensitivity analysis confirms the importance of considering all the main drivers of ZIKV transmission, i.e., environmental suitability, socioeconomic risk of exposure to mosquitoes, and intensity of case importations, to quantitatively assess the risk of local ZIKV transmission in the contiguous US.

Figure S5: The cumulative risk map of local ZIKV transmission for each county in the contiguous US of counterfactual scenario 2, where the temperature settings across the contiguous US are set to be the same as that in Miami-Dade, Florida. The color scale indicates for any given county the probability of experiencing at least one ZIKV outbreak with more than 20 infections.

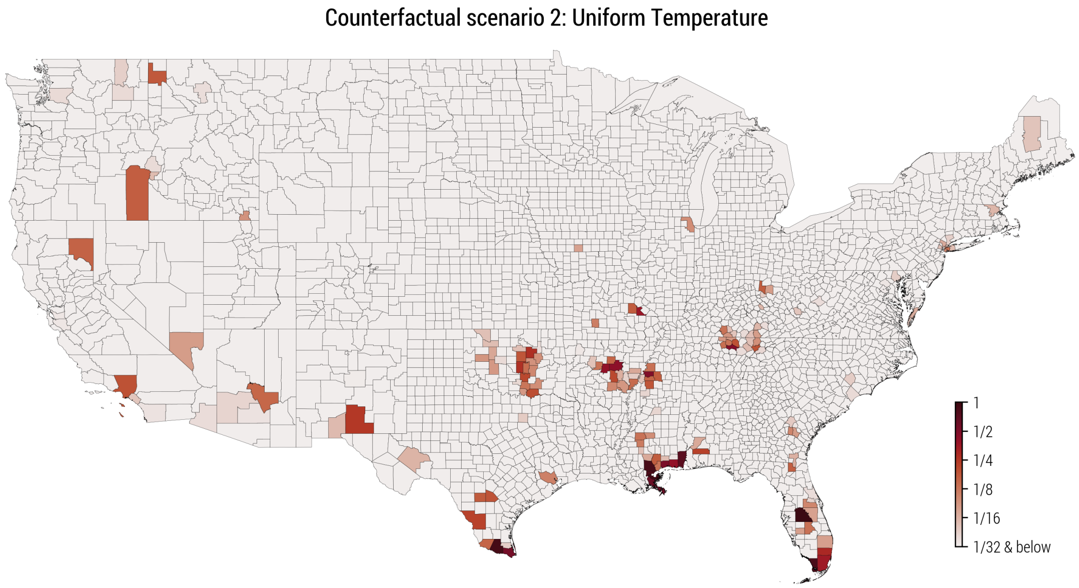

Figure S6: The cumulative risk map of local ZIKV transmission for each county in the contiguous US of counterfactual scenario 3, where the socioeconomic risks of exposure to mosquitos across the contiguous US are set to be the same as that in Miami-Dade, Florida. The color scale indicates for any given county the probability of experiencing at least one ZIKV outbreak with more than 20 infections.

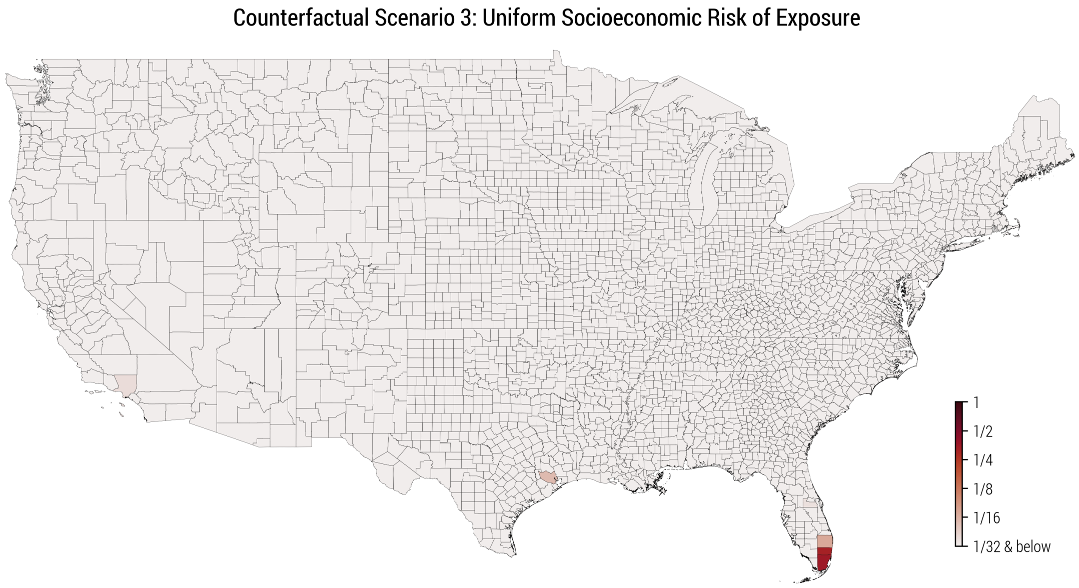

References

[1] Duygu Balcan, Bruno Gonçalves, Hao Hu, José J Ramasco, Vittoria Colizza, and Alessandro Vespignani. Modeling the spatial spread of infectious diseases: The GLobal Epidemic and Mobility computational model. *Journal of Computational Science*, 1(3):132–145, 2010.

[2] Isaac I Bogoch, Oliver J Brady, MU Kraemer, Matthew German, Marisa I Creatore, Manisha A Kulkarni, John S Brownstein, Sumiko R Mekaru, Simon I Hay, Emily Groot, et al. Anticipating the international spread of Zika virus from Brazil. *The Lancet*, 387(10016):335–336, 2016.

[3] Oliver J Brady, Peter W Gething, Samir Bhatt, Jane P Messina, John S Brownstein, Anne G Hoen, Catherine L Moyes, Andrew W Farlow, Thomas W Scott, and Simon I Hay. Refining the global spatial limits of dengue virus transmission by evidence-based consensus. *PLOS Neglected Tropical Diseases*, 6(8):e1760, 2012.

[4] Centers for Disease Control and Prevention. Estimated range of Aedes aegypti and Aedes albopictus in the United States, 2017, 2017.

[5] Gerardo Chowell, Doracelly Hincapie-Palacio, Juan Ospina, Bruce Pell, Amna Tariq, Sushma Dahal, Seyed Moghadas, Alexandra Smirnova, Lone Simonsen, and Cécile Viboud. Using Phenomenological Models to Characterize Transmissibility and Forecast Patterns and Final Burden of Zika Epidemics. 2016.

[6] CIESIN-Columbia University. Global Population Count Grid Time Series Estimates, 2017.

[7] Mark R Duffy, Tai-Ho Chen, W Thane Hancock, Ann M Powers, Jacob L Kool, Robert S Lanciotti, Moses Pretrick, Maria Marfel, Stacey Holzbauer, Christine Dubray, et al. Zika virus outbreak on Yap Island, federated states of Micronesia. *New England Journal of Medicine*, 360(24):2536–2543, 2009.

[8] Nuno R Faria, Josh Quick, IM Claro, Julien Theze, Jacqueline G de Jesus, Marta Giovanetti, Moritz UG Kraemer, Sarah C Hill, Allison Black, Antonio C da Costa, et al. Establishment and cryptic transmission of Zika virus in Brazil and the Americas. *Nature*, 546(7658):406, 2017.

[9] Nuno Rodrigues Faria, Raimunda do Socorro da Silva Azevedo, Moritz UG Kraemer, Renato Souza, Mariana Sequetin Cunha, Sarah C Hill, Julien Thézé, Michael B Bonsall, Thomas A Bowden, Ilona Rissanen, et al. Zika virus in the Americas: early epidemiological and genetic findings. *Science*, 352(6283):345–349, 2016.

[10] Neil M Ferguson, Zulma M Cucunubá, Ilaria Dorigatti, Gemma L Nedjati-Gilani, Christl A Donnelly, Maria-Gloria Basáñez, Pierre Nouvellet, and Justin Lessler. Countering the Zika epidemic in Latin America. *Science*, 353(6297):353–354, 2016.

[11] Michael A. Johansson, Ann M. Powers, Nicki Pesik, Nicole J. Cohen, and J. Erin Staples. Nowcasting the spread of chikungunya virus in the Americas. *PLOS ONE*, 9(8):e104915, aug 2014.

[12] Matt J Keeling and Pejman Rohani. *Modeling infectious diseases in humans and animals*. Princeton University Press, 2008.

[13] Moritz UG Kraemer, Marianne E Sinka, Kirsten A Duda, Adrian Mylne, Freya M Shearer, Oliver J Brady, Jane P Messina, Christopher M Barker, Chester G Moore, Roberta G Carvalho, et al. The global compendium of Aedes aegypti and Ae. albopictus occurrence. *Scientific Data*, 2:150035, 2015.

[14] Moritz UG Kraemer, Marianne E Sinka, Kirsten A Duda, Adrian QN Mylne, Freya M Shearer, Christopher M Barker, Chester G Moore, Roberta G Carvalho, Giovanini E Coelho, Wim Van Bortel, et al. The global distribution of the arbovirus vectors Aedes aegypti and Ae. albopictus. *Elife*, 4:e08347, 2015.

[15] Adam J. Kucharski, Sebastian Funk, Rosalind M. Eggo, Henri-Pierre Mallet, W. John Edmunds, and Eric J. Nilles. Transmission Dynamics of Zika Virus in Island Populations: A Modelling Analysis of the 2013–14 French Polynesia Outbreak. *PLoS Neglected Tropical Diseases*, 10(5):e0004726, may 2016.

[16] Adam J. Kucharski, Sebastian Funk, Rosalind M. Eggo, Henri-Pierre Mallet, W. John Edmunds, and Eric J. Nilles. Transmission Dynamics of Zika Virus in Island Populations: A Modelling Analysis of the 2013–14 French Polynesia Outbreak. *PLOS Neglected Tropical Diseases*, 10(5):e0004726, may 2016.

[17] Louis Lambrechts, Krijn P Paaijmans, Thanyalak Fansiri, Lauren B Carrington, Laura D Kramer, Matthew B Thomas, and Thomas W Scott. Impact of daily temperature fluctuations on dengue virus transmission by Aedes aegypti. *Proceedings of the National Academy of Sciences*, 108(18):7460–7465, 2011.

[18] Andrew J Monaghan, Cory W Morin, Daniel F Steinhoff, Olga Wilhelmi, Mary Hayden, Dale A Quattrochi, Michael Reiskind, Alun L Lloyd, Kirk Smith, Chris A Schmidt, et al. On the seasonal occurrence and abundance of the Zika virus vector mosquito Aedes aegypti in the contiguous United States. *PLOS Currents*, 8, 2016.

[19] Erin A Mordecai, Jeremy M Cohen, Michelle V Evans, Prithvi Gudapati, Leah R Johnson, Catherine A Lippi, Kerri Miazgowicz, Courtney C Murdock, Jason R Rohr, Sadie J Ryan, et al. Detecting the impact of temperature on transmission of Zika, dengue, and chikungunya using mechanistic models. *PLOS Neglected Tropical Diseases*, 11(4):e0005568, 2017.

[20] W. D Nordhaus. Geography and Macroeconomics: New Data and New Findings. *Proceedings of the National Academy of Sciences*, 103(10), 20180102 2005.

[21] W. D. Nordhaus and X. Chen. Global Gridded Geographically Based Economic Data (G-Econ), Version 4. 20180102 2016.

[22] OAG, Aviation Worlwide Limited. OAG, connecting the world of travel, 2017.

[23] T Alex Perkins, Amir S Siraj, Corrine W Ruktanonchai, Moritz UG Kraemer, and Andrew J Tatem. Model-based projections of Zika virus infections in childbearing women in the Americas. *Nature Microbiology*, 1:16126, 2016.

[24] Paul Reiter, Sarah Lathrop, Michel Bunning, Brad Biggerstaff, Daniel Singer, Tejpratap Tiwari, Laura Baber, Manuel Amador, Jaime Thirion, Jack Hayes, Calixto Seca, Jorge Mendez, Bernardo Ramirez, Jerome Robinson, Julie Rawlings, Vance Vorndam, Stephen Waterman, Duane Gubler, Gary Clark, and Edward Hayes. Texas Lifestyle Limits Transmission of Dengue Virus. *Emerging Infectious Diseases*, 9(1):86–89, jan 2003.

[25] Steven P Russell, Kyle R Ryff, Carolyn V Gould, Stacey W Martin, and Michael A Johansson. Detecting Local Zika Virus Transmission In The Continental United States: A Comparison Of Surveillance Strategies. *bioRxiv*, page 145102, 2017.

[26] Daouda Sissoko, Amrat Moendandze, Denis Malvy, Claude Giry, Khaled Ezzedine, Jean Louis Solet, and Vincent Pierre. Seroprevalence and risk factors of chikungunya virus infection in Mayotte Indian Ocean, 2005-2006: a population-based survey. *PLOS ONE*, 3(8):e3066, aug 2008.

[27] C. J. Willmott and K. Matsuura. Terrestrial Air Temperature: 1900-2010 Gridded Monthly Time Series, 2001.

[28] Qian Zhang, Kaiyuan Sun, Matteo Chinazzi, Ana Pastore y Piontti, Natalie E Dean, Diana Patricia Rojas, Stefano Merler, Dina Mistry, Piero Poletti, Luca Rossi, et al. Spread of Zika virus in the Americas. *Proceedings of the National Academy of Sciences*, 114(22):E4334–E4343, 2017.
